# Alterations of PTEN and SMAD4 methylation in diagnosis of breast cancer: implications of methyl II PCR assay

**DOI:** 10.1186/s43141-021-00154-x

**Published:** 2021-04-06

**Authors:** Menha Swellam, Entsar A. Saad, Shimaa Sabry, Adel Denewer, Camelia Abdel Malak, Amr Abouzid

**Affiliations:** 1 Biochemistry Department, Genetic Engineering and Biotechnology Research Division, High Throughput Molecular and Genetic Laboratory, Center for Excellences for Advanced Sciences, National Research Centre, Dokki, Giza, Egypt; 2 Chemistry Department, Faculty of Science, Damietta University, Damietta, 34517 Egypt; 3 Surgical Oncology Department, Mansoura Oncology Centre, Faculty of Medicine, Mansoura University, Mansoura, Egypt

**Keywords:** Breast cancer, DNA methylation, Circulating molecular marker, Diagnosis

## Abstract

**Background:**

Diagnosis of breast cancer is more complicated due to lack of minimal invasive biomarker with sufficient precision. DNA methylation is a promising marker for cancer diagnosis. In this study, authors evaluated methylation patterns for PTEN and SMAD4 in blood samples using EpiTect Methyl II QPCR assay quantitative PCR technology.

**Results:**

Methylation status for PTEN and SMAD4 were statistically significant as breast cancer patients reported hypermethylation compared to benign and control groups (77.1 ± 17.9 vs. 24.9 ± 4.5 and 15.1 ± 1.4 and 70.1 ± 14.4 vs. 28.2 ± 0.61 and 29.5 ± 3.6, respectively). ROC curve analysis revealed that both PTEN (AUC = 0.992) and SMAD4 (AUC = 0.853) had good discriminative power for differentiating BC from all non-cancer individuals (benign and healthy combined) compared to routine tumor markers CEA (AUC = 0.538) and CA15.3 (AUC = 0.686). High PTEN methylation degree was associated with late stages (84.2 ± 17.4), positive lymph node (84.2 ± 18.5), positive ER (81.3 ± 19.7), positive PgR (79.5 ± 19.1), and positive HER2 (80.7 ± 19.0) vs. 67.4 ± 13.8, 70.6 ± 14.8, 72.8 ± 14.9, 72.5 ± 14.7, and 70.2 ± 13.5 in early stages, negative lymph node, negative ER, negative PgR, and negative HER2, respectively. Similar results were obtained regarding SMAD4 methylation. Sensitivity, specificity, positive and negative predictive values, and accuracy for methylated PTEN were 100%, 95%, 99.1%, 100%, and 95%, respectively when differentiated BC from all-non cancer controls. Interestingly, PTEN could distinguish early BC stages with good sensitivity 84.4%, 51.4%, 69.1%, 72%, and 70%, respectively.

**Conclusion:**

Methylation status of PTEN and SMAD4 is a promising blood marker for early detection of breast cancer. Future studies are needed for their role as prognostic markers.

## Background

Breast cancer (BC) is the most widely neoplasm in women [1] that progresses silently, leading to neighboring metastasis and poor prognosis. Moreover classification of breast cancer into relevant molecular subtypes (luminal A, luminal B, triple-negative/basal-like, HER2-enriched, and normal-like breast cancer) is important in treatment strategy and hence their prognosis [2]. The survival rate improves with early diagnosis [3]. Numerous hazard factors such as family history, gene mutations, sex, aging, estrogen, and unhealthy lifestyle could increase BC probability [4]. The most BC widely recognized symptom is a new mass or lump [5] that has been diagnosed by many methods such as screening tests (physical tests and mammogram), lab tests (tumor markers as CEA and CA15.3), and breast biops y[6]. However, these screening methods have limitations such as radiation exposure and maybe affected by breast density [7]. Moreover, no effective blood-based marker is available for BC detection, particularly for patients with early-stage and low-grade tumor [7].

Epigenetic mutations, involved DNA methylation, were responsible for the control of gene expression by regulating gene transcription [8]. Most DNA methylation is catalyze by DNA methyltransferases and exhibits essential physiological role in numerous key functions of cells [9]. Aberrant promoter methylation of numerous tumor suppressor genes was obtained in BC initiator lesions, representing that DNA methylation is an early event in breast carcinogenesis [10].

Among these tumor suppressor genes, tensin homolog (PTEN) gene and its function loss affects the increased levels of tumor aneuploidy that is one of the most projecting features of human cancers [11]. PTEN gene is reported to be mutated in a number of human malignancies including prostate [12], cervical [13], and BCs [14]. Former studies have shown an association of PTEN promoter methylation with its diminished expression in BC [15] which may indicate its role in breast carcinogenesis [16]. In a previous study [17], it was reported that transforming growth factor β (TGF-β) facilitates PTEN suppression in pancreatic cancer cell. Another tumor suppressor gene is SMAD4 which shows critical role in the TGF-β signaling pathway as well. TGF-β acts as significant characters in various biological procedures, such as apoptosis, in addition to cancer initiation and progression [18, 19]. Mutation and promoter hypermethylation of SMAD4 has been reported in numerous human cancers [20].

Thus, the two suppressor genes PTEN and SMAD4 are linked through TGF-β pathway as they can mediate carcinogenesis [21], and their loss may attributes to cancer aggressiveness [22]. Hence, this study aimed to investigate methylation patterns of both PTEN and SMAD4 among primary BC patients and compare their role with other non-cancer groups as well as assess their diagnostic effectiveness with other tumor markers (CEA and CA15.3). Also, we aimed to evaluate the association between these patterns and clinicopathological features.

## Methods

### Patients and specimen collection

A total of 110 patients were involved; they were enrolled from Surgical Department, Oncology Centre, Egypt. They were divided according to their clinical diagnosis into primary BC group (*n* = 80, 72.7%) and benign breast diseases (*n* = 30, 27.2%). BC patients fulfilled the inclusion criteria as being diagnosed with primary BC patients before receiving any treatment or underwent surgical resection and have not any other tumors. Demographic and clinico–pathological data for enrolled individuals were collected from their medical reports. BC patients were diagnosed according to their staging and grading systems following the TNM classification [23]. In addition, 30 healthy individuals were included and they served as control group. Blood samples (3 mL) were withdrawn from all participants in tubes EDTA and centrifuged at 4000 rpm for 5 min. at 4 °C for further processing to detect the investigated markers.

### Hormonal receptor status analysis and molecular subtype of BC

For each sample, the expression of hormonal receptors and BC molecular subtype was identified by immunohistochemistry (IHC) method using formalin-fixed paraffin-embedded tissue. IHC staining was evaluated. Both ER and PgR were consider positive if ≥ 10% nuclei was positively stained using ten high-power field, and HER-2neu was considered positive if scored as + 3.

### Tumor markers assessment

By enzyme-linked immunosorbent assay (ELISA), both CEA and CA15.3 were determined in serum samples using available commercial kits (Immuno-speccorporation, the Netherlands) based on instructions in the manual manufacturer’s protocol. Their levels were detected using GloMax-Multi detection system (Promega, USA).

### DNA extraction

DNA was extracted using QIAamp DNA Min kit (Qiagen, Hilden, Germany), according to the manufacturers’ recommendations, which based on spin column for DNA extraction method. The purity and the concentration of the extracted DNA were detected before further investigation using nano-drop spectrophotometer (Quawell, Q-500, Scribner, USA) then stored at – 20 °C till further restriction digestion.

### Detection of DNA methylation pattern

DNA methylation pattern was detected using EpiTect Methyl II quantitative polymerase chain reaction (qPCR) System (Qiagen, Germany). This technique is based on the detection of remaining input genome after digestion with a methylation-sensitive restriction enzyme. This technique requires 2 phases: phase I, digestion of DNA samples which was carried out following the manufacturer instruction of EpiTect Methyl II DNA Restriction Kit. Briefly, it was as follows: the input genomic DNA was aliquoted into four equal portions and subjected to four tubes mock (M0), methylation-sensitive (Ms), methylation-dependent (Md), and methylation-sensitive-dependent enzyme (Msd). All 4 reactions were incubated at 37 °C for 6 h then at 65 °C for 20 min using thermal cycler (SureCycler 8800, Agilent, Santa Clara, CA, USA). Then, phase II is as follows: qPCR for methylation status (using Max3005P QPCR system; Stratagene, Agilent Technologies, CA, USA), the enzyme reactions were mixed directly with qPCR master mix (RT^2^ qPCR SYBR Green/ROX Master Mix, Cat number 330520) and were dispensed into a PCR plate containing pre-aliquoted primer mixes (EpiTect Methyl II qPCR Primer Assay). Real-time PCR is carried out using specified cycling conditions, 95 °C for 10 min (1 cycle), then 99 °C for 30 s and 72 °C for 1 min (3 cycles), and finally 97 °C for 15 s and 72 °C for 1 min (40 cycles). Finally, the raw ΔCT values were pasted into the data analysis spreadsheet (EpiTect Methyl II PCR Array Microsoft Excel based data analysis template), which automatically calculates the relative amount of methylated and unmethylated DNA fractions.

### Data analyses

Data were analyzed using SPSS (SPSS, Inc., Chicago, USA; version 20) and GraphPad prism (version 6). Significant difference between groups was determined using (ANOVA) for normally distributed values and Kruskal-Wallis for non-normal distributed variables and Fisher’s LSD as post-hoc test and *P* value were two-tailed and considered significant if < 0.05. Receiver operating characteristic (ROC) curve was constructed among studied groups to identify the specificities and sensitivities for investigated genes. Person correlation coefficient (*r*) was utilized for determining the association between target gene methylation degree and other parameters.

## Results

### Patients characteristic

Patients demographic and clinicopathological data were summarized in Table 1. Both benign and healthy controls were age matched (*P =* 0.923) with BC patients. Most of BC patients (56.2%) and patients with benign breast disease (56.7%) were premenopausal. For benign breast lesion, they were pathologically divided into 12 with follicular hyperplasia, 10 with intraductal papillomatosis, and 8 with fibrocystic changes. According to the hormonal receptor status, BC patients were classified into luminal A (ER/PR^+^, HER2^−^; 8/80 (10%)), luminal B (ER/PR^+^, HER2^+^; 52/80 (65%)), and triple negative (ER^−^/PR^−^/HER2^−^; 20/80 (25%)).

**Table 1:** Demographic and clinicopathological data of breast carcinoma patients

Parameter	Breast cancer	Benign	Healthy	***P*** value
**Number**	80	30	20	–
**Age (years, mean** ± **SD)**	52.4 ± 9.1	52.0 ± 9.3	51.5 ± 8.9	0.923^a^
**Menopause, number (percentage)**				
Post-menopausal	35 (43.8%)	13 (43.3%)	8 (40%)	0.955^b^
Pre-menopausal	45 (56.2%)	17 (56.7%)	12 (60%)	
**Tumor invasion number (percentage)**				
In situ	32(40%)	–	–	–
Invasive	48(60%)	–	–	–
**Tumor depth (stage)**				
Early (T ≤ 2)	35(43.8%)	–	–	–
Late (T > 2)	45 (56.2%)	–	–	–
**Tumor grade**				
Low (G1)	30(37.5%)	–	–	–
High (G2–3)	50 (62.5%)	–	–	–
**Lymph node invasion**				
Negative	42 (52.5%)	–	–	–
Positive	38 (47.5%)	–	–	–
**Estrogen receptor (ER)**				
Negative	40 (50%)	–	–	–
Positive	40 (50%)	–	–	–
**Progesterone receptor (PgR)**				
Negative	28 (35%)	–	–	–
Positive	52 (65%)	–	–	–
**HER-2/neu status**				
Negative	28 (35%)	–	–	–
Positive	52 (65%)	–	–	–

*Statistical analysis was carried out by (a) ANOVA test and (b) chi-squared (*χ*^2^) test in case of dichotomized values

*P* > 0.05 is considered non-significant; *P* ≤ 0.05 is considered significant

### DNA methylation status and tumor markers level among investigated groups

**Fig. 1:**
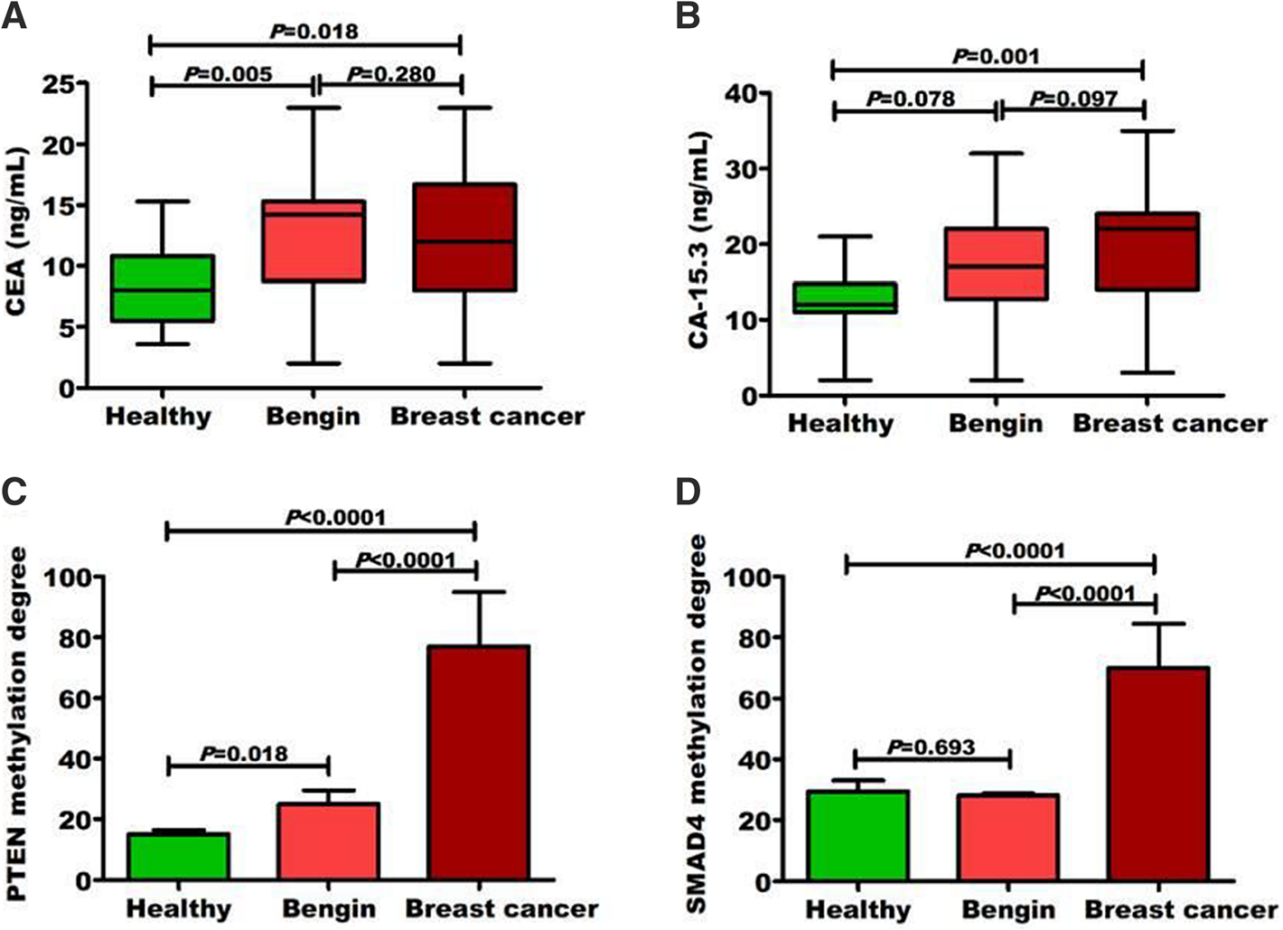
The distribution of investigated markers among the investigated groups. **a** Level of CEA, (**b**) level of CA 15.3, (**c**) level of PTEN, and (**d**) level of SMAD4

**Fig. 2:**
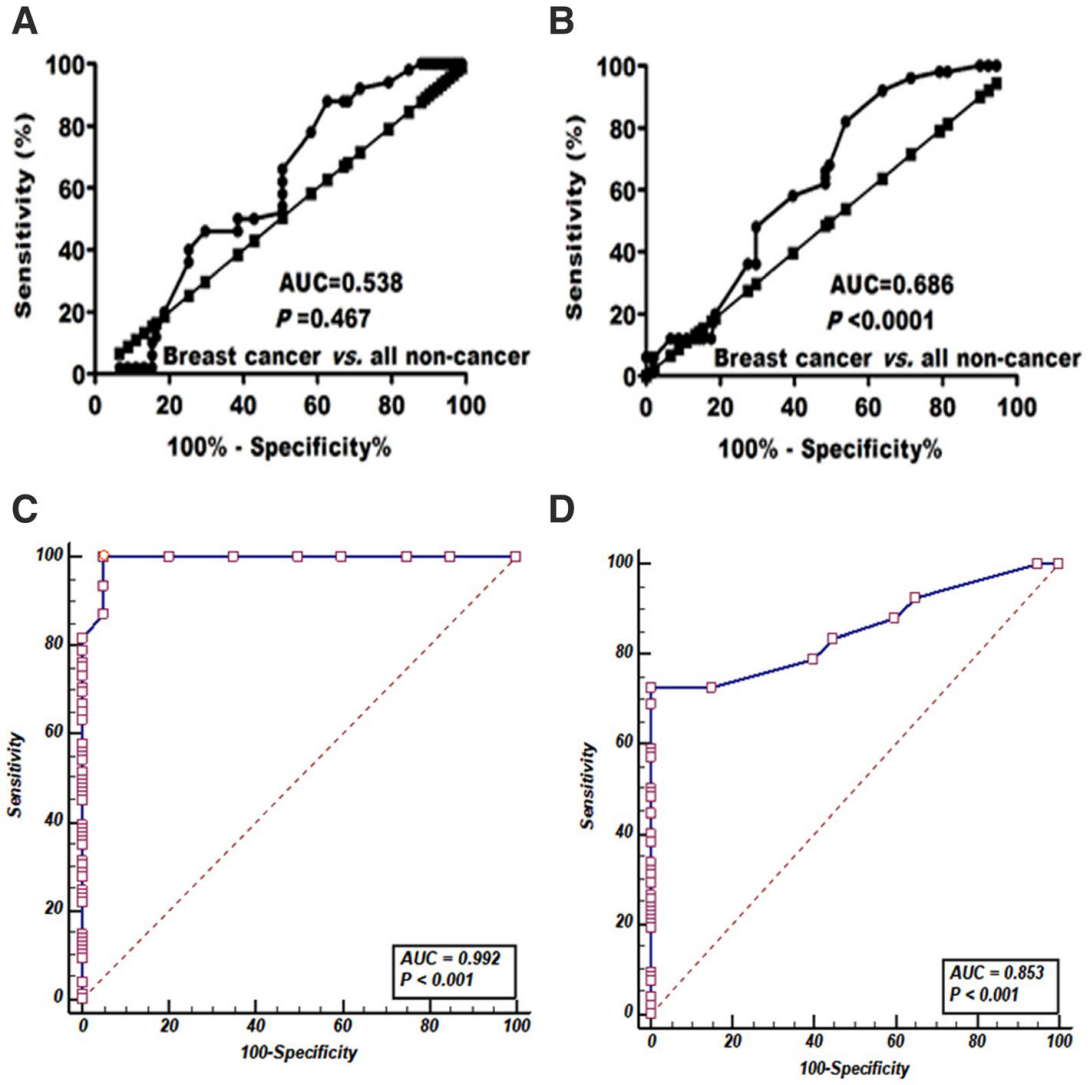
Receiver operating characteristic curve for (**a**) CEA, (**b**) CA15.3, (**c**) methylated PTEN, and **d** methylated SMAD4 to distinguish patients with breast cancer from non-cancer (healthy and benign combined)

BC patients were associated with high methylation degree of PTEN and SMAD4. In contrast to CEA (Fig. 1a) and CA15.3 (Fig. 1b), BC patients were associated with high methylation degree of PTEN (77.1 ± 17.9 vs. 24.9 ± 4.5 for benign and 15.1 ± 1.4 for healthy individuals; Fig. 1c) and SMAD4 (70.1 ± 14.4 vs. 28.2 ± 0.61 for benign and 29.5 ± 3.6 for healthy individuals; Fig. 1d). To evaluate the diagnostic efficacy for investigated items, ROC curves (Fig. 2a–d) were plotted. Despite CEA (AUC = 0.538) and CA15.3 (AUC = 0.686), ROC curve analysis revealed that both PTEN (AUC = 0.992) and SMAD4 (AUC = 0.853) had extremely discriminative power for good differentiating BC from all non-cancer individuals (benign and healthy combined).

### PTEN and SMAD4 high methylation pattern and clinicopathological features

**Fig. 3:**
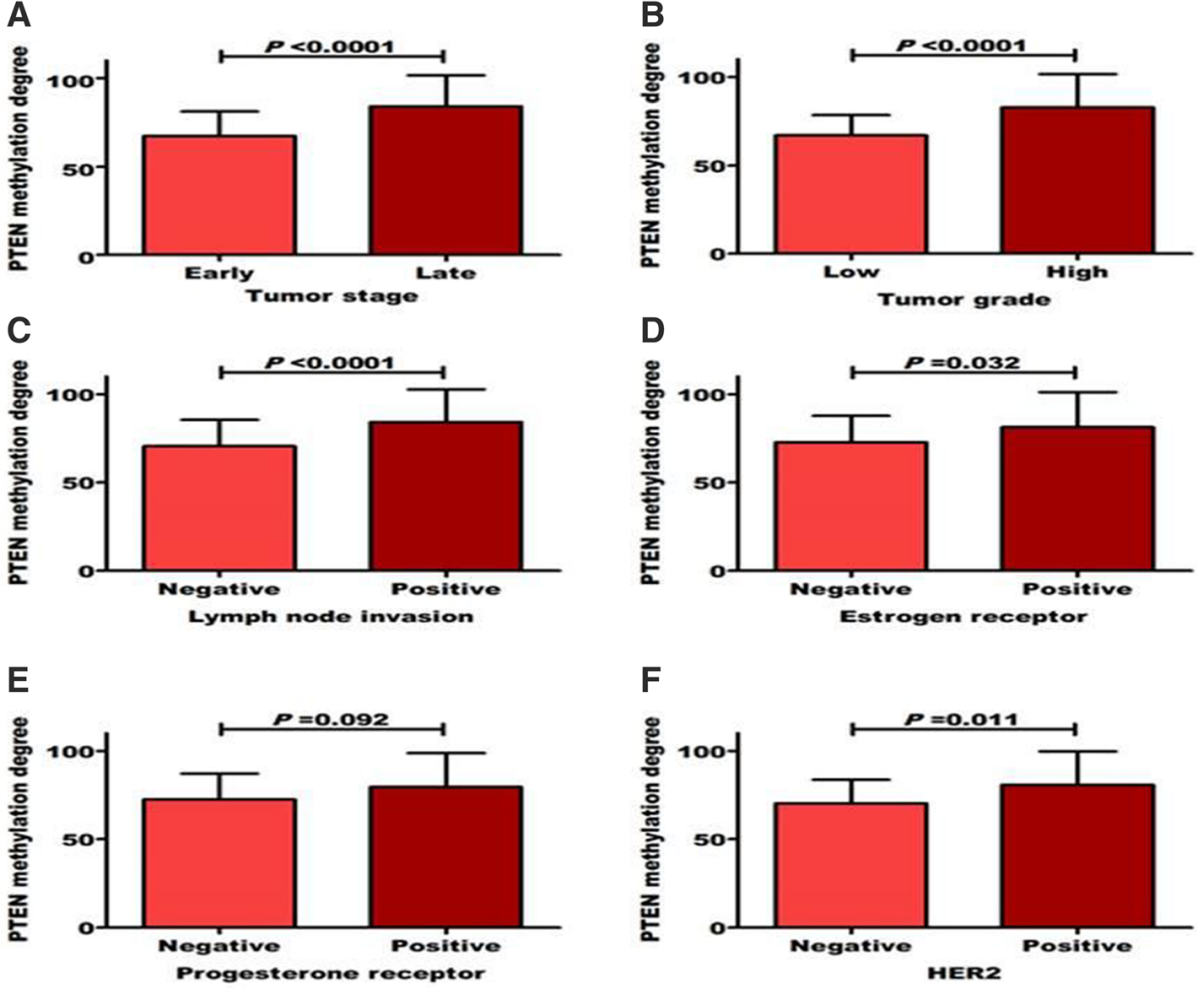
Distribution of PTEN methylation degree among breast cancer patients according to tumor (**a**) stages, (**b**) grades, (**c**) lymph node, (**d**) estrogen receptor, (**e**) progesterone receptor, and (**f**) HER2 statuses. Significant difference was determined using Student’s *t* test. *P* value < 0.05 was considered significant

**Fig. 4:**
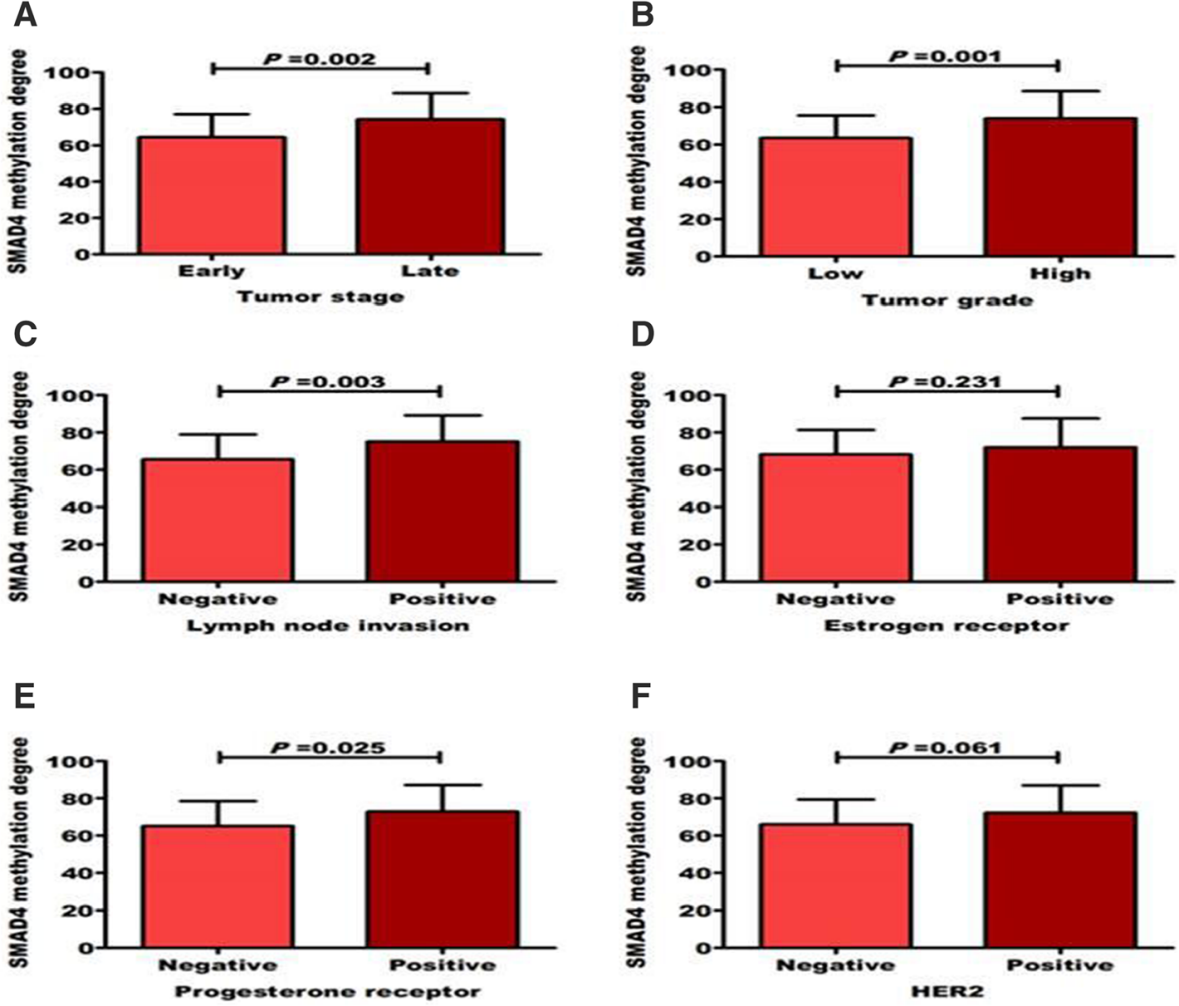
Distribution of SMAD4 methylation degree among breast cancer patients according to tumor (**a**) stages, (**b**) grades, (**c**) lymph node, (**d**) estrogen receptor, **e** progesterone receptor, and (**f**) HER2 statuses. Significant difference was determined using Student’s *t* test. *P* value < 0.05 was considered significant

No significant result was reported between methylation of PTEN and SMAD4 with either age or menopausal status as shown in Table 2. High PTEN methylation degree was significantly associated with tumor late stage (84.2 ± 17.4 vs*.* 67.4 ± 13.8), positive lymph node metastasis (84.2 ± 18.5 vs. 70.6 ± 14.8), positive ER (81.3 ± 19.7 vs. 70.6 ± 14.8), positive PR (79.5 ± 19.1 vs. 72.5 ± 14.7), and positive HER2 (80.7 ± 19.0 vs*.* 70.2 ± 13.5) as shown in Fig. 3a–f. Similarly, high SMAD4 methylation degree was significantly associated (*P* < 0.05) with high grade (74 ± 14.4 vs. 63.3 ± 12.01), tumor late stage (84.2 ± 17.4 vs. 64.5 ± 12.7), positive lymph node metastasis (75.1 ± 14.1 vs. 65.6 ± 13.4), positive ER (72 ± 15.5 vs. 68.2 ± 13.2), positive PR (72.8 ± 14.5 vs. 65.2 ± 13.3), and positive HER2 (72.3 ± 14.6 vs. 66 ± 13.4) as shown in Fig. 4a–f. Moreover, as shown in Table 2, both methylated PTEN and SMAD4 genes were significantly (*P* < 0.05) correlated with CA15.3, tumor stage, grade, and lymph node involvement. Moreover, PTEN methylation was significantly related with breast cancer subtypes. High significant correlation was detected between PTEN and SMAD4 methylation (*r* = 0.727, *P* = 0.0001).

**Table 2:** Correlation between methylated PTEN and SMAD4 with other parameters

Factors	PTEN methylation status		SMAD4 methylation status	
	*R*	*P* value	*R*	*P* value
**Age**	− 0.09	0.315	− 0.017	0.844
**Menopause**	− 0.1	0.270	− 0.016	0.861
**CEA**	0.209	0.017	0.086	0.329
**CA15-3**	0.395	0.0001	0.265	0.002
**Tumor stage**	0.507	0.0001	0.320	0.004
**Tumor grade**	0.492	0.0001	0.329	0.003
**Lymph node**	0.454	0.0001	0.323	0.004
**ER**	0.203	0.07	0.120	0.289
**PgR**	0.315	0.004	0.237	0.034
**HER2**	0.042	0.711	0.194	0.084
**Molecular subtypes**	0.321	0,0001	0,154	0.175
**Methylated SMAD4**	0.727	0.0001		

*Correlations were determined according to Pearson coefficient; *P* < 0.05 was significant

### Diagnostic performance of PTEN and SMAD4 methylation degree

As reported in Table 3, methylated PTEN and SMAD4 genes were superior to CEA and CA15.3 in early BC detection. Also, their diagnostic ability was better than other tumor marker in differentiated early stages and low-grade breast cancer; however, the sensitivity of CEA was more than that of SMAD4 in detection of low-grade tumors (Table 3).

**Table 3:** Overall sensitivity, specificity, PPV, NPV, and accuracy of investigated methylated genes and tumor markers

Variables	Sen.%	Spe. %	PNV%	NPV%	Acc.%
**Early detection of breast cancer vs. all non-cancer**					
CEA (ng/ml)	70	40	65.1	45.5	58.5
CA15.3 (ng/ml)	75	48	69.8	54.5	64.6
PTEN	100	94	96.4	100	97.7
SMAD4	100	100	100	100	100
**Early stage breast cancer vs. all non-cancer**					
CEA (ng/ml)	51.1	51.4	57.5	45	51.3
CA15.3 (ng/ml)	51.1	51.4	57.5	45	51.3
PTEN	84.4	51.4	69.1	72	70
SMAD4	66.7	40	58.8	48.3	55
**Low-grade breast cancer vs. all non-cancer**					
CEA (ng/ml)	66	60	73	51.4	66
CA15.3 (ng/ml)	54	50	54	50	54
PTEN	80	70	80	70	80
SMAD4	60	60	60	60	60

## Discussion

BC is the most frequently occurring in women worldwide and the second most prevalent cancer [24]. Also, it is the most common tumor affecting Egyptian females [25]. Early-stage cancer detection may minimize death rates for BC. There are multiple diagnostic methods for breast tumor detection; however, because of the weakness of these approaches, researchers have used distinctive biomarkers as alternatives to detect BC [26]. Carcinogenesis arises from significant modifications in DNA methylation within the cell. These alterations are distinguished by the hypomethylation or hypermethylation of multiple 5′-cytosine-phosphate-guanine-3′ (CpG) islands [27]. So, the aim of this study was to evaluate whether the methylation pattern of both SMAD4 and PTEN genes was correlated with clinical variables of breast carcinoma.

Both SMAD4 and PTEN have been revealed a closely linked in the regulation of tumor infiltration and distant metastasis. For instance, there were findings suggest that the loss of Smad4 and PTEN may lead to more aggressive disease and poor prognosis in patients with colorectal adenocarcinoma compared to the loss of SMAD4 or PTEN alone [28], pancreatic cancer [29], lung cancer [30], and invasive intestinal-type gastric cancer [22]. PTEN is a tumor suppressor gene; promoter methylation of PTEN is implicated in various types of cancer including non-small-cell lung carcinoma [31], gastric [32], cervical cancer [13, 33], and endometrial carcinoma [34], including BC [14, 15, 35]. Promoter hypermethylation of the PTEN gene is a key event in the progression of BC [36]. In the current study, it was observed that there were no correlations between age of individuals and methylation degree of PTEN (*P =* 0.315); this result was in harmony with the study by [16] Alam et al., and it was in dissimilarity with a study of Kazim et al. [37], who revealed a significant difference with north Indian breast cancer patient’s age. In study by Wu et al. [35], who used MSP technology, only a few of CpG islands in the promoter region of genes were stately. This study noticed that hypermethylation of *PTEN* was significantly associated with menopausal status (*P* = 0.027); this is in disagreement with current results that investigated non-significant difference with menopause status (*P =* 0.238); variability in results could be due to the size of study groups, as well as applied methodology. For example, Kazim et al. used another method (methylation-specific PCR) on another race (Asian populations) [37].

Methylated PTEN status reported a significant correlation with tumor markers, tumor stage, and tumor grade; also, there was a significant correlation between methylated PTEN and PR (*P =* 0.004). This in agreement with the study of Alam et al. [16], as there was significant correlation noticed among PTEN methylation and tumor grade and tumor stage. These findings were in consistent with previously reported studies such as Yari [36] and Zhang et al. [14], as they indicated no association between methylated PTEN with other pathological characteristics of patients. This difference in results may be due to genetic predisposition. Further study is in progress to investigate the relation between PTEN and clinical characteristics on a large cohort of Egyptian patients. Breast cancer can be divided into four molecular subtypes that differ in their response as previously reported [2]. In the current study, a significant relation was reported between PTEN and molecular subtypes of breast cancer which reveal the impact for detection of methylation status for further treatment strategy [38].

SMAD4 is a tumor suppressor gene that regulates cell proliferation, distinction, and extracellular matrix production [39]. It has been reported that SMAD4 acts as a prognostic biomarker in various human malignancies such as colorectal carcinoma [40] and pancreatic carcinoma [41], but its role as a prognostic marker in breast carcinoma is still unclear [42]. Deckers et al. [43] informed that SMAD4/TGF-β-induced growth protection and apoptosis only happened at early stages of BC. However, Li et al. [44] noticed that SMAD4 stimulated apoptosis in estrogen receptor-α (ERα)-positive BC cells.

In a few cancer types, promoter hypermethylation of the SMAD4 gene has been recorded but does not appear to be a common event in carcinogenesis [20]. In contrast, hypermethylation was not determined in colorectal malignancies and small intestinal neuroendocrine cancers [45–47]. In the current study, methylation SMAD4 pattern was significantly correlated with CA15.3, tumor stage and grade, and lymph node. These investigations about methylation status of SMAD4 in our study were the first study reporting implication of the methylated SMAD4 in Egyptian breast cancer patients.

Moreover, a significant correlation was reported between methylation status of both PTEN and SMAD4 (*P* = 0.0001), and this could be attributed that both are tumor suppressor genes related to TGF-β pathway [17, 22]. In this study, methylation of SMAD4 can distinguish breast cancer patients from healthy and benign individuals, where methylation degree of SMAD4 in breast cancer is higher than methylation in benign and healthy controls. Methylated SMAD4 pattern may be a good power for early detection of BC and even when considering the high-risk BC group. This in disagreement with several studies that revealed that hypermethylation was not found in colorectal malignancies and small intestinal neuroendocrine tumors [45–47], but our results were in agreement with study by Onwuegbusi et al. who reported that 70% of esophageal carcinoma cases were found to be hypermethylated at SMAD4 promoter [48].

## Conclusions

In conclusion, to our knowledge, this is the first study to focus on the diagnostic efficacy of the methylation pattern for both SMAD4 and PTEN in BC and to assess their correlation with each other as two important tumor suppressor genes related to certain pathways (TGF-β, tyrosine kinase). Moreover, their superiority in detection of BC over routine classical tumor markers (CEA and CA15.3) as serum-based marker maybe directed to a better diagnostic marker for BC. Hence, a further study on large cohort samples is recommended.

## Data Availability

Data is not available due to [ethical/legal/commercial] restrictions: Due to the nature of this research, participants of this study did not agree for their data to be shared publicly, so supporting data is not available.

## Abbreviations

BC: Breast cancer
PTEN: Phosphatase and tensin homolog
TGF-β: β tumor growth factor
qPCR: Quantitative polymerase chain reaction
